# Oral Cancer Screening: Past, Present, and Future

**DOI:** 10.1177/00220345211014795

**Published:** 2021-05-26

**Authors:** S. Warnakulasuriya, A.R. Kerr

**Affiliations:** 1 King’s College London and WHO Collaborating Centre for Oral Cancer, London, UK; 2 Department of Oral and Maxillofacial Pathology, Radiology & Medicine, New York University College of Dentistry, New York, NY, USA

**Keywords:** cancer risk, mouth neoplasms, oral potentially malignant disorders, mass screening, clinical oral examination, case finding

## Abstract

Oral cancer is a major public health problem, and there is an increasing trend for oral cancer to affect young men and women. Public awareness is poor, and many patients present with late-stage disease, contributing to high mortality. Oral cancer is often preceded by a clinical premalignant phase accessible to visual inspection, and thus there are opportunities for earlier detection and to reduce morbidity and mortality. Screening asymptomatic individuals by systematic visual oral examinations to detect the disease has been shown to be feasible. A positive screen includes both oral cancer and oral potentially malignant disorders. We review key screening studies undertaken, including 1 randomized clinical trial. Screening of high-risk groups is cost-effective. Strengths and weaknesses of oral cancer screening studies are presented to help guide new research in primary care settings and invigorated by the prospect of using emerging new technologies that may help to improve discriminatory accuracy of case detection. Most national organizations, including the US Preventive Services Task Force, have so far not recommended population-based screening due a lack of sufficient evidence that screening leads to a reduction in oral cancer mortality. Where health care resources are high, opportunistic screening in dental practices is recommended, although the paucity of research in primary care is alarming. The results of surveys suggest that dentists do perform oral cancer screenings, but there is only weak evidence that screening in dental practices leads to downstaging of disease. Where health care resources are low, the feasibility of using primary health care workers for oral cancer screening has been tested, and measures indicate good outcomes. Most studies reported in the literature are based on 1 round of screening, whereas screening should be a continuous process. This review identifies a huge potential for new research directions on screening for oral cancer.

## Introduction

Recent global estimates indicate that cancers of the lip and oral cavity (referred to here as “oral cancers”) collectively represent the 16th most common malignant neoplasm worldwide, with almost 355,000 new incident cases per year (Miranda-Filho and Bray 2020). Greater than 90% of oral cancers are squamous cell carcinomas and two-thirds of cases occur in developing countries, half of which are in South Asia. India alone accounts for approximately 100,000 incident cases annually. On average, the rates for men are currently twice as high as for women, although there are exceptions, such as in Taiwan, where the male/female ratio is 10:1. The risk of developing oral cancer increases with age, and most cases occur in people over the age of 50 y. There are also wide geographical variations in incidence, with Papua New Guinea estimated as having the highest rate of oral cancer in the world. Other areas characterized by high incidence rates for oral cavity cancers are found in the South Asia (e.g., Maldives, Sri Lanka, India, and Pakistan), East Asia (e.g., Taiwan), parts of Western Europe (e.g., northeastern France and Portugal) and Eastern Europe (e.g., Hungary, Slovakia, and Slovenia), and parts of Latin America and the Caribbean (e.g., Brazil, Uruguay, and Puerto Rico) (Warnakulasuriya and Greenspan 2020). Oral cancer is linked to social and economic status and deprivation, with the highest rates occurring in the most disadvantaged sections of the population (Warnakulasuriya and Greenspan 2020).

Treatment of patients with early stage oral cancer indicates that these patients have a good prognosis (Seoane et al. 2012) and improved rates of survival and quality of life. However, early stage cancers are often asymptomatic and mimic benign conditions, reducing the likelihood for the public to seek care, and therefore screening provides an opportunity for early detection. The aim of this critical review is to present the current evidence on the state of art on screening for oral cancer, to stimulate future research, policy development, and appropriate strategies to reduce deaths and suffering from oral cancer worldwide. This article consists of sections on organized programs, evaluation of their validity, adjunctive techniques, use of primary health care workers (PHCWs), e-health and mobile technology for screening, screening for human papillomavirus (HPV), predicting cancer risk models, and recommendation statements.

## Principles of Screening

Screening has been defined as “the identification of unrecognised disease by the application of a test to people who are asymptomatic, in order to identify those who probably have the disease and to distinguish them from those who probably do not.” The criteria dictating whether a disease is “screenable” were established by Wilson and Jungner (1968), and these criteria have been expanded by national bodies (e.g., UK National Screening Committee 2003), based on new evidence and to reduce harm from screening (Appendix Table 1). Screening should be distinguished from case finding. *Case finding* is the term used for patients who present with abnormal signs or symptoms and undergo a diagnostic test to establish a diagnosis, in contrast to screening, which is applied to asymptomatic patients. The 2 principal benefits of cancer screening are to both “down-stage” the disease and achieve a reduction in mortality (and morbidity). In the case of oral cancer, in which the majority have a premalignant phase, screening criteria are designed to capture patients with oral cancer and oral potentially malignant disorders (OPMDs), a group of disorders with an increased risk for oral cancer (Warnakulasuriya et al. 2020). This expands the purpose of oral cancer screening to not only detect oral cancer earlier but also detect and manage those patients with OPMDs who are at risk for developing cancer.

The conventional test applied in most screening studies and programs involves a systematic visual inspection and palpation of the oral cavity under a bright light source to detect abnormal oral findings that raise the index of suspicion for oral cancer or OPMDs, as well as evaluation of the neck for any enlarged lymph nodes consistent with regional metastasis. This screening test is referred to as the visual oral examination (VOE). A screening test should have the ability to select all cases with the disease among the screened population.

It is important to note that a screening test is not intended to be diagnostic but aims to capture patients with such abnormal oral findings and to accelerate the referral and application of more specific diagnostic procedures by a specialist (i.e., reexamination and, if deemed necessary, diagnostic testing by tissue biopsy followed by definitive histopathological diagnosis).

## Organized Oral Cancer Screening Programs

An organized screening program consists of several essential elements, including high attendance rates, good calibration of screeners, quality control of the applied test, and availability of a referral pathway for detected cases to receive adequate treatment. These elements allow quality control, monitoring of the process, and evaluation of outcomes. Several screening models have been applied by various researchers, including population-based screening (both by home visits or by invitation to attend screening events), opportunistic screening at dental practices, integrating oral cancer screening with general health screening, screening at the place of work (e.g., industrial sites), or self-screening. Risk-based modeling to screen “at-risk” populations would seem to offer greater efficiency compared to the general population–based screening employed by most studies. In Table 1 (and Appendix Table 2), we provide examples of oral cancer screening models undertaken in different countries, provide a critique, and offer recommendations for improvements. While most of these programs were studies to assess the logistical feasibility, reproducibility, or accuracy of the screening test in a specific health system, only 3 programs have been conducted to assess the impact to health and are discussed below.

**Table 1.** A Critique of Reported Oral Cancer Screening Models.

Screening Model	Critique	Recommendations
Population screening by home visits versus invitation	Studies reporting house-to-house visits reported greater coverage and good compliance to screening (95%–98%) (India, Sri Lanka).	A social marketing campaign could increase compliance.
		Provide repeated screening at suitable intervals.
	Poor compliance to invitational screening (United Kingdom, Japan). Selection bias is a serious weakness.	Develop risk prediction models to preferentially screen “at-risk populations.”
	Low compliance to attend a referral center for confirmation of diagnosis attenuates benefits of the program (52% in the Sri Lanka study).	Use mobile technology to take and send clinical images of screen-positive patients to experts for quick consultations.
	Most studies do not incorporate a risk prediction model to identify and screen “at-risk” patients.	Develop artificial intelligence to analyze clinical images generated during a screening.
	Most studies did not provide a series of multiple screenings at regular intervals.	Use mobile screening units that can travel from village to village.
Integrated with medical screeningOpportunistic screening	Reduces the cost of the program.The project would need coordination to integrate with medical screeners.Largely performed in dental offices and not in other primary care settings.	To increase yield, integrate with screenings for tobacco/alcohol-related disorders.
		Provide appropriate training, especially for oral cavity cancer, to increase accuracy.
		Strengthen undergraduate curricula on oral cancer detection (dental, medical, nursing, and other allied health care training programs).
	A workforce is available but needs additional training; cost neutral.	
	No benefit to people with poor access to care or those who attend primary care clinics irregularly.	Develop tool kits and e-learning modules to train screeners.
		National practice-based networks should be established for data collection and future research.
		Develop risk prediction models for primary care to assess risk profile.
High-risk screening	Provides the best cost effectiveness.	Combine with risk factor health promotion and treatment programs to achieve compliance.
	Poor compliance (Italy).	
Industrial/workplace	Most reported studies are on white-collar workers.	Dentists working in industries to receive Continuing Professional Development packages on oral cancer screening
	Compliance is better than in other models	
Mouth self- examination (MSE)	High negative predictive value.Leaflets are inadequate in instructing how to perform MSE.	Visual media (instead of printed leaflets) may improve accuracy.
	High volume of self-referrals to specialist centers.	MSE to be demonstrated at dental visits by auxiliaries.

**Figure 1.**
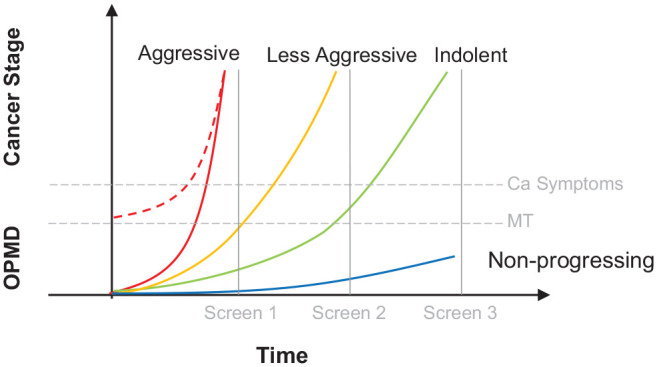
Length-time bias. Four different scenarios are depicted. “Aggressive” oral cavity SCCs can arise de novo (broken red line) or develop from OPMDs. They progress rapidly (hence steep curve) and are unlikely to be detected in an asymptomatic state during screening. “Less aggressive” oral squamous cell carcinomas (OSCCs) may develop from OPMDs. They progress less rapidly (hence less steep curve) and can be detected as asymptomatic OSCCs during screening. “Indolent” OSCCs develop from longer-standing OPMDs. They progress slowly (hence the flatter curve) but do eventually transform. “Nonprogressing” OPMDs never transform. These scenarios portray length-time bias: patients with aggressive OSCCs have a short potential screening window and are less likely to be captured by a screening program. Patients with slower-growing OSCCs have a longer potential screening window and are more likely to be detected when they are asymptomatic. As a result, a higher proportion of slower-growing OSCCs is found in the screened group, causing an apparent improvement in survival. Different risk stratification analyses are needed for OPMDs detected by screening. Repeated screening at intervals allows for a better understanding of the natural history. ca, cancer; MT, malignant transformation; OPMD, oral potentially malignant disorder. This figure is available in color online.

**Figure 2.**
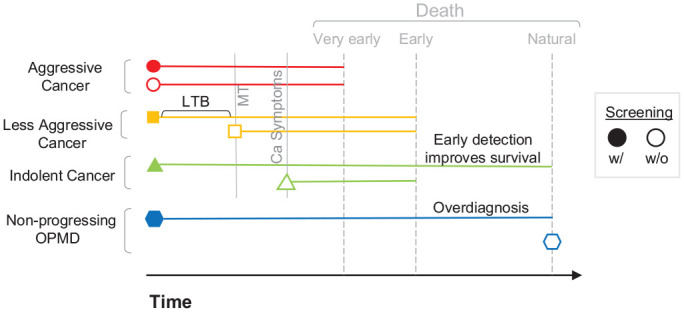
Lead time bias/overdiagnosis. The same 4 scenarios are depicted differently. Aggressive oral squamous cell carcinomas (OSCCs) are not affected by screening, and patients all die very early, irrespective of screening. “Less aggressive” OSCCs are detected earlier by screening, but this has no impact on survival and represents lead-time bias, an illusion that those who are screened live longer with the cancer. “Indolent” OSCCs detected earlier by screening positively influence survival. Patients who are not screened die early, and those who are screened if appropriately treated early do not die of cancer but of “natural” causes. This exemplifies the value of screening programs. Patients with “nonprogressing” OPMDs who are not screened die of “natural causes” with undetected OPMDs. This is an example of overdiagnosis bias. In reality, the natural history of cancer development from OPMDs and the aggressiveness of OSCCs is highly variable and unpredictable, and the relative contribution of lead-time and overdiagnosis bias remains to be elucidated across populations. LTB, lead time bias; OPMD, oral potentially malignant disorder.

Cuba was the first country to introduce a national oral cancer case-finding program dating back to 1982 (Fernández Garrote et al. 1995; Santana et al. 1997). The main objective was to improve the stage at which cases were detected without waiting for patients to present with symptoms. Subjects recruited were those who presented to a dental office with dental problems and underwent VOE. Despite the use of the term *case finding* in the project title, this project could be construed as “opportunistic” screening. Nevertheless, between 1982 and 1990, over 10 million people were examined, of whom 0.3% were “screen positive,” although the referral compliance for expert examination was poor (29%). A favorable stage shift was reported with an increase in cancers detected at stage 1 and a reduction in advanced cancers. Sixteen percent of 4,412 incident oral cancers recorded in Cuba during the time period were identified through screening. A case control study within the Cuban study suggests that screening can reduce the risk of advanced stage disease (Sankaranarayanan et al. 2002), but a program review could not identify any reduction in incidence or mortality from oral cancer since the introduction of the Cuban program (reporting ended in 1997). Our imprecise understanding of the natural history of oral cavity cancer and, in particular, OPMDs suggests caution when interpreting reduction in “stage shifts” and survival. The impact of lead time, length time, and overdiagnosis biases (Figs. 1, 2) must be considered, and screening studies exploring mortality as the primary end point are critical (Patz et al. 2000).

Only 1 randomized control trial (RCT) for oral cancer screening has been reported. It was conducted in Kerala, India, during 1994 to 2009. The trial was planned to test whether oral cancer screening could reduce mortality among the screened population, a critical end point to assess the impact of any screening study. Over a 15-y period, there were 4 rounds of screening, and overall, 87,655 (91% of the target population) were screened at least once. After 3 rounds of screening, the authors reported a significant 34% reduction in oral cancer mortality among a high-risk group of tobacco and/or alcohol users (Sankaranarayanan et al. 2005). After 4 rounds of screening, the authors’ final report indicated a sustained reduction in mortality at 81% (95% confidence interval [CI], 69%–89%) and, furthermore, a 38% (95% CI, 8%–59%) reduction in the incidence of oral cancer in the screened population compared with a control population (Sankaranarayanan et al. 2013). Their report highlights the life-saving benefits of the program to high-risk subjects detected with oral cancer or OPMDs and far outweighed any potential harm to those subjects who were screened and rescreened as negative. The significant findings of this RCT are widely acknowledged. Cochrane reviews, however, found a number of methodological weaknesses in that there was lack of allocation concealment and the small numbers of clusters randomized, which increase the potential for imbalance across the trial groups. There were also variations in risk factors between the 2 arms at baseline, which might have confounded the data, and the close proximity of clusters in the 2 arms could have led to contamination (Brocklehurst et al. 2013).

More recently, a national oral cancer screening program undertaken in Taiwan has, to some extent, substantiated the findings of the Kerala study (Chuang et al. 2017). Between 2004 and 2009, over 2 million Taiwanese adults who were smokers and/or betel quid chewers were invited for a biennial oral examination by a dentist or a trained physician. Fifty-five percent attended for screening and 4,110 were confirmed to have oral cancer at their first screen. The program was evaluated by comparing screening data and outcomes between the screened population and those who refused screening. Cancer registry statistics were used to obtain follow up data on the nonscreened group. There was evidence of a stage shift, with 46.5% in stages I and II in the screened group compared with 39.6% in the nonscreened group (and a 21% reduction in stage III or IV oral cancer diagnoses in the screened group). There was also a 26% reduction in mortality in the screened group (relative risk [RR], 0.74; CI, 0.72–0.77) and a reduction in incidence of oral cancer in subsequent screens (133.4 per 100,000 compared with 190.9 per 100,00 in the nonscreened group). Taiwan is the only country in the world to initiate a sustained national oral cancer screening program. Screening is currently offered to high-risk groups, that is, betel quid chewers (including ex-chewers) and smokers. This was the first study to use risk-stratification modeling to target high-risk patients.

As demonstrated in a Japanese oral cancer screening program, screening should not be limited to a one-off program but should be repeated to benefit the population who receive screening (Nagao et al., 2000). The study demonstrated that new OPMDs can be detected by an annual screening.

Whether screening is a cost-effective strategy in oral cancer detection has been addressed by economic evaluations of the Kerala and Taiwan studies. We summarize their findings in Appendix Table 3 along with some modeling studies (e.g., Speight et al. 2006) that have investigated costs and benefits of screening. Several of these studies report that screening, especially in an opportunistic setting and directed at high-risk groups, could be cost-effective.

## Evaluation of the Validity of Screening Programs

A paramount factor for assessing the success of a screening program is the accuracy and validity of index test performance. Screening test accuracy is evaluated by a number of measures, including sensitivity, specificity, and predictive values. Screen positives should be validated against an appropriate gold standard (e.g., clinical diagnosis by an expert and/or a definitive histopathological diagnosis) to calculate true- and false-positive rates. A random sample of “screen-negative” subjects should be rescreened to calculate the true- and false-negative rates. Unfortunately, not all oral cancer screening studies reported have been assessed in this way. A meta-analysis of 7 reported studies (published up to 1997) has been performed, updating an earlier review in 2002 (Downer et al. 2004). Three of these studies were reported from Asia using PHCWs to perform screening, 1 was undertaken in Japan using general dentists, and the other 3 were UK studies conducted by specialists. Although there was significant heterogeneity among these studies, visual screening had acceptable discriminatory ability to detect target disease. Among 16 European oral cancer screening studies evaluated by us, only 6 studies had reported such analysis (Warnakulasuriya et al. 2015).

A Cochrane systematic review of test accuracy of 10 screening studies found similar variability in sensitivity (0.50–0.99) but a consistently high value for specificity—greater than 0.80 (Walsh et al. 2013). In Table 2, we provide an update on all eligible studies published until 2020. Variations of sensitivity and specificity noted among various programs are mainly due to differences in settings and manpower employed for these screening studies. These analyses suggest that screeners are more adept in pronouncing a subject as a true screen negative for OPMDs or oral cancer than categorizing subjects as a true screen positive. While it is encouraging that the specificity-related performance of the VOE is high (largely related to the fact that most patients in a screening trial have a completely normal examination), the underlying heterogeneity of sensitivity across these studies is of concern and may be explained by the inherent challenge of the screener being able to differentiate OPMDs and oral cancer from benign “lookalike” conditions. In the Kerala study, despite 4 cycles of screening, the reported sensitivity of the visual examination in detecting OPMDs and oral cancer was 67.4% (188/279).

**Table 2.** Evaluation of Screening Programs That Used Visual Oral Examination as a Screening Test.

Country	No. Screened	% Positive	Sensitivity	Specificity	PPV	NPV	Reference
Sri Lanka	29,295	4.2	0.95	0.81	0.58	0.98	Warnakulasuriya et al. (1984)
India	39,331	1.3	0.59	0.98	0.31	0.99	Mehta et al. (1986)
Sri Lanka	57,124	6.2	0.97	0.75	0.80	0.95	Warnakulasuriya and Nanayakkara (1991)
United Kingdom	2,027	2.7	0.74	0.99	0.67	0.99	Jullien et al. (1995)
Japan	802	9.7	0.60	0.94	0.67	0.96	Ikeda et al. (1995)
India	2,069	10.3	0.94	0.98	0.87	0.99	Mathew et al. (1997)
Japan	19,056	4.1	0.92	0.64	0.78	0.86	Nagao et al. (2000)
United Kingdom	309	5.5	0.71	0.99	0.86	0.98	Downer et al. (2004)
Portugal	727	3.4	0.96	0.98	0.96	0.98	Monteiro et al. (2015)
Sri Lanka	685	11.3	0.63	0.82	—	—	Amarasinghe et al. (2016)
Taiwan	13,878	5.2	0.99	0.99	0.62	0.99	Chuang et al. (2017)
Brazil	359	1.1	0.83	0.95	—	—	Simonato et al. (2019)
India	3,445	1.2	0.82	0.98	0.83	0.98	Birur et al. (2019)

NPV, negative predictive value; PPV, positive predictive value.

## Evaluation of Current and Emerging Adjunctive Techniques

The use of commercially available adjunctive techniques (i.e., aids to enhance or improve the accuracy of the VOE) would seem logical. In this technology age, it is conceivable that one day, the VOE will be replaced. Such screening adjuncts involve “wide-field” evaluation of the oral cavity beyond the naked eye, employ light-based technologies or oral rinses (or feasibly both), and are designed to accurately detect and delineate abnormal mucosal “fields” that equate with oral carcinogenesis. Research on commercially available adjunctive techniques for the detection of oral cancer or OPMDs (i.e., optical-based adjuncts, vital staining, and cytopathologic platforms) relates to their diagnostic accuracy compared to gold-standard histopathology following detection of OPMDs by VOE (Lingen, Tampi, et al. 2017). Tissue autofluorescence devices and vital rinsing with toluidine blue have been explored in a screening setting, although no platform has, as yet, demonstrated convincing evidence to support their utility (Su et al. 2010; Truelove et al. 2011; Simonato et al. 2019).

Several wide-field optical imaging technologies have been researched and were reviewed in this journal (Ilhan et al. 2020). Their advantage is that they are noninvasive and point-of-care. None of these technologies have been validated prospectively in large screening studies, and practical issues such as cost and convenience likely would abrogate use across primary care settings. Optical imaging technologies often require complex analysis, and with the advent of artificial intelligence, the ability for technologies to make accurate clinical decisions in the field is a possibility.

Saliva contains biological molecules reflective of a number of human disease processes, and the term *salivaomics* encompasses an array of potential biomarkers based on salivary genomics/epigenomics, proteomics, transcriptomics, metabolomics, and microbiomics (Wong 2012). The anatomic proximity of saliva to oral cavity and oropharynx cancers, coupled with the simplicity of saliva or oral rinse collection (i.e., “liquid biopsy”), supports the feasibility of using this biofluid for oral cancer screening. Research to detect candidate salivary biomarkers has exploded over the past few years, and studies largely involve testing single or combinations of putative biomarkers in case-control studies. These studies have been systematically reviewed elsewhere (Gualtero and Suarez Castillo 2016; Assad et al. 2020; Li et al. 2020), and while there are some encouraging findings, methodological issues and the inherent heterogeneity of oral cavity carcinogenesis limit their interpretation. The use of multiplex panels of salivary biomarkers might mitigate the issue of disease heterogeneity. If validated, salivary tests might also have benefit of being self-administered for “home screening.” Compared to salivary markers, there are fewer studies exploring serum- or plasma-based markers for oral cavity cancer (Guerra et al. 2016), and despite similar performance in case-control studies, the screening of blood samples for a cancer that is in direct contact with the saliva seems less appealing. Yet, panels of salivary or blood-based diagnostic panels that can simultaneously detect cancer signatures across multiple organ systems would seem to be the ultimate goal. Potential inflammatory plasma protein biomarkers of patients with oral squamous cell carcinomas have been reported (Liu et al. 2018).

Two commercial point-of-care “salivary” diagnostic platforms claim to predict the presence or absence of oral cavity cancer. One platform measures soluble CD44 and total protein content of oral rinses with a reported sensitivity/specificity of 90%/62%, respectively (Franzmann and Donovan 2018), and the other examines 6 salivary messenger RNA (mRNA) markers (IL-1β, IL-8, OAZ1, SAT1, S100P, and DUSP1) (Martin 2016). Neither platform is approved by the US Food and Drug Administration for population or opportunistic screening. They are marketed for triaging patients with OPMDs, and both require validation in future studies by other research groups.

In summary, there are currently no screening adjunctive techniques that have been prospectively tested in oral cancer screening trials in primary care. Novel tests using salivary and serum are in development. However, they have disadvantages such as costs, equipment, and lack of qualified professionals in low-income countries.

## Use of PHCWs for Oral Cancer Screening in Low- and Middle-Income Countries

In high-income countries like the United States, the role of the dental team in opportunistic screening cannot be underestimated (Psoter et al. 2019). However, due to the relatively low percentage of medical and dental clinicians in low/middle-income countries, several population-based oral cancer screening programs have used PHCWs. In fact, this modeling accounts for approximately a third of all reported oral cancer screening studies (see Appendix Table 2). Data suggest that PHCWs with some training were able to screen for oral cancer and OPMDs with good accuracy, similar to trained dental practitioners. For low- and middle-income countries with a high incidence of oral cancer, challenged resources, and a limited dental workforce, PHCWs seem suited to this task (Downer et al. 2004). A recent review discusses the strengths and weaknesses of these models (Nagao and Warnakulasuriya 2020). Data from some of these studies are highlighted in Table 2. In general, PHCW models have served communities for various diseases where there is fewer than 1 physician per 1,000 people, which is the minimal threshold advised by the World Health Organization. In a systematic review citing 156 studies using PHCWs for delivery of basic health care for noncommunicable diseases including screening, the authors suggest 6 key lessons: 1) select qualified PHCWs embedded within the community they serve; 2) provide detailed, ongoing training and supervision; 3) authorize them to prescribe medications and render autonomous care; 4) equip them with reliable systems to track patient data; 5) furnish them consistently with medications and supplies; and 6) compensate them adequately, commensurate with their roles (Heller et al. 2019). Applying these lessons might improve the delivery of oral cancer screening.

## E-health and Mobile Technology for Screening

The use of mobile technology by PHCWs to improve screening services has an inherent appeal for application in remote areas. Mobile phone applications have been developed and piloted with PHCWs for oral cancer screening in India (Birur et al. 2019), in Corboda (Argentina), and more recently in Malaysia (Haron et al. 2021). These applications allow transmission of oral images deemed as “screen positive” to a “remote” specialist. In the Indian study, PHCWs screened 3,445 industrial workers and sent images of lesions and normal mucosa for each subject. In total, 11.4% were deemed screen positive by the PHCWs. In addition to the remote specialist, the study design also included an onsite specialist. Of the screen positives by the PHCWs, 15.3% and 17.5% were deemed false positive and 0.03% and 0.2% were deemed false negative by the remote and onsite specialists, respectively. These studies are promising.

## Screening for HPV-Positive Oropharyngeal Cancer

Given the global increases in HPV-driven oropharynx cancers (Kreimer et al. 2020), in which the conventional oral examination is limited by the anatomic location (i.e., tonsils), the saliva or expectorated oral rinses to screen for oncogenic HPV are particularly attractive. In a preliminary study, oral rinses to detect HPV-16 DNA and mRNA in HPV-positive oropharynx cancer patients demonstrated moderate to poor sensitivity (D’Souza et al. 2019). However, serum antibodies to HPV-16 E6 demonstrated high sensitivity, and a similar finding was reported in a second study (Lang Kuhs et al. 2016). A recent screening study of HPV-16 DNA in 665 “cancer-free” subjects (employing both saliva and oral rinse sampling) identified 9 HPV-positive individuals who were followed and retested every 3 to 6 mo. Three subjects with persistent HPV-16 infection >30 mo were evaluated by an otolaryngologist, leading to the identification of HPV-positive oropharynx cancer in 1 subject (Tang et al. 2020). The feasibility of performing a risk-based opportunistic screening for oral HPV infection in dental offices has been tested (Rindal et al. 2019). In this study, subjects presenting for routine dental evaluation took a short screening questionnaire to assess risk for prevalent oral HPV infection. Those meeting a risk threshold performed an oral rinse that was evaluated for oncogenic HPV subtypes.

## Predicting Cancer Risk Models

The Harvard Cancer Risk Index was developed to predict individual cancer risk for major cancers in the United States (Colditz et al. 2000). The index offers a simple estimation of personal risk of cancer based on lifestyle and may help identify “at-risk populations,” allowing for primary or secondary prevention. Colditz et al. (2000) did not include oral cancer in their analysis. Risk modeling has been incorporated into both lung and breast cancer screening (Katki et al. 2016; Cintolo-Gonzalez et al. 2017). Screening for oral cavity cancer/OPMDs using risk prediction models is considered cost-effective (Speight et al. 2006). A risk prediction model has been developed for head and neck cancers (including oral cancer) based on age, gender, race/ethnicity, education level, and cigarette smoking/alcohol consumption (Lee et al. 2020). This modeling does not take OPMDs into account, and a group in Sri Lanka developed a risk model for OPMDs based on field surveys on lifestyles and their association with OPMDs (Amarasinghe et al. 2010). A risk prediction model that includes age, sex, race, smoking, alcohol use, lifetime sexual partners, and oncogenic HPV status has been developed for future screening for oropharyngeal cancers in the United States (Tota et al 2019).

## Recommendation Statements from National Bodies

Several national organizations have evaluated the available evidence on oral cancer screening and published clinical guidelines whether to screen or not to screen for oral cancer/OPMDs. Based on the evidence available up to 2015, the UK National Screening Committee recommended against screening for oral cancer and is currently under rereview (UK National Screening Committee 2020). The committee based this recommendation on the evidence that 1) only a small percentage of OPMDs progressed to malignancy, 2) it was unclear which individuals with OPMDs progressed to oral cancer, 3) there was insufficient evidence to determine the accuracy of screening tests in the general UK population, and 4) it was not clear which individuals detected through screening should be offered treatment. The committee reaffirmed an earlier report that effectiveness of early treatment for oral cancer in leading to better outcomes than late treatment had been established.

The US Preventive Services Task Force (USPSTF) also concluded that the available evidence was insufficient to assess the balance of benefits and harms of screening for oral cancer in asymptomatic adults (Moyer 2014). Counterarguments to the USPSTF have been proposed (Edwards 2013). The recommendation was intended for primary medical care providers and did not pertain to dental providers or otolaryngologists, who, it was conceded, may conduct a comprehensive examination of the oral cavity during a clinical encounter.

The American Cancer Society recommends that adults aged 20 y or older who have periodic health examinations should have the oral cavity examined as part of a cancer-related checkup. The American Dental Association recommends that clinicians perform a VOE in all adult patients during initial, routine, or emergency visits (Lingen, Abt, et al. 2017).

## Conclusions

Screening for oral cancer has been researched using several models. It is important to select the best model that suits a particular population based on the disease incidence, available resources, and the health system of the country. Screening studies performed to date demonstrate potential strengths and weaknesses but are useful to provide a general framework to help inform clinicians and policy makers when considering recommendations for oral cancer screening. We outline some challenges and offer solutions for future research. Screening high-risk populations or introducing telemedicine for consultation with specialists may reduce costs and increase efficiency. Combining adjunctive aids to enhance visual examination or using salivary/blood-based testing using proven biomarkers has not been investigated in primary care and could be incorporated into future oral cancer screening research programs.

## Author Contributions

S. Warnakulasuriya, contributed to conception, design, data acquisition, analysis, and interpretation, drafted and critically revised the manuscript; A.R. Kerr, contributed to design, data acquisition, analysis, and interpretation, drafted and critically revised the manuscript. Both authors gave final approval and agree to be accountable for all aspects of the work.

## Supplemental Material

sj-pdf-1-jdr-10.1177_00220345211014795 – Supplemental material for Oral Cancer Screening: Past, Present, and Future Click here for additional data file. Supplemental material, sj-pdf-1-jdr-10.1177_00220345211014795 for Oral Cancer Screening: Past, Present, and Future by S. Warnakulasuriya and A.R. Kerr in Journal of Dental Research
