# Supplementary material for: Oral Cancer Screening: Past, Present, and Future
Source: J Dent Res. 2021 May 26;100(12):1313–20. doi: 10.1177/00220345211014795 (PMC8529297; doi:10.1177/00220345211014795)
Supplement: sj-pdf-1-jdr-10.1177_00220345211014795 – Supplemental material for Oral Cancer Screening: Past, Present, and Future [file sj-pdf-1-jdr-10.1177_00220345211014795.pdf]

Supplemental Table 1: Wilson and Jungner Criteria for Screening (1968) vs UK National Screening Criteria (2003)

**Wilson and Jungner criteria for screening**	**UK National Screening Committee criteria**
1.The condition to be screened for should be an important health problem<br>2.There should be an accepted and effective treatment for patients with recognized disease<br>3. Facilities for diagnosis and treatment of those screened positive should be available<br>4. The disease should have a recognizable latent or early symptomatic stage<br>5. There should be a suitable test or examination<br>6. The test should be acceptable to the population<br>7. The natural history of the condition, including development from latent to declared disease, should be adequately understood<br>8. There should be an agreed policy on who should be offered treatment and the appropriate treatment to be offered<br>9. The programme should be cost-effective<br>10. Screening should be a continuing process and not a 'once and for all'	**The Condition:**<br>1. Must be an important health problem<br>2. The epidemiology and natural history must be understood, and there must be a detectable latent asymptomatic or early symptomatic phase<br>3. All cost-effective primary prevention interventions should have been implemented where possible<br>**The Test:**<br>4. Should be simple, safe, and validated<br>5. The distribution of test values should be known (e.g., sensitivity and specificity), and the criteria for a positive test should be agreed upon<br>6. Should be acceptable to the population<br>7. There should be an agreed-upon policy and process for the further referral and diagnostic investigation of individuals who test positive<br>**The Treatment:**<br>8. Should be an effective treatment or intervention for patients found to have disease and evidence that this early treatment leads to better outcomes<br>9. Should be evidence-based policies covering which individuals should be offered treatment and the appropriate treatment to be offered<br>10. Clinical management of the condition and patient outcomes should be optimized<br>**The Screening program:**<br>11. There must be evidence from randomized clinical trials (RCTs) that the screening program is effective in reducing mortality or morbidity<br>12. Should be clinically, socially, and ethically acceptable<br>13. Benefit should outweigh any physical or psychological harm<br>14. Must be cost effective<br>15. There must be a clear plan for managing the programme and agreed-upon quality assurance standards<br>16. There must be adequate staffing and facilities for the program, and for referrals, diagnosis, and treatment<br>17. All other options for managing the condition should have been considered<br>18. Evidence-based information explaining the positive and negative aspects of the program<br>must be available to participants<br>19. Screening intervals, eligibility for screening and the testing process should be scientifically justifiable to the public

Supplemental Table 2. Examples of screening studies related to reported oral cancer screening models.

Screening Model	First Author/Year (Country)
Population screening by home visits vs invitation	Axéll 1976 (Sweden)<br>Warnakulasuriya et al. 1984 (Sri Lanka)<br>Mehta et al. 1986 (India)<br>Warnakulasuriya and Nanayakkara 1991 (Sri Lanka)<br>Jullien et al. 1985(UK)<br>Matthew et al 1997 (India)<br>Sankaranarayanan et al. 2000 (India)<br>Monteiro et al. 2015 (Portugal)<br>Chuang et al. 2017 (Taiwan)
Integrated with medical screening	Dombi et al. 1994 (Hungary)<br>Nagao et al 2000 (Japan)
Opportunistic screening	Fernandez Garrote et al. 1995 (Cuba)<br>Lim et al. 2003 (UK)<br>Chang et al. 2011(Taiwan)<br>Psoter et al. 2019 (US)
High risk screening	Talamini et al 1994 (Italy)<br>Harris et al. 2004 (UK)
Industrial/workplace	Fever 1997 (UK)<br>Nagao et al 2003 (UK)<br>Warnakulasuriya et al. 2010 (India)
Mouth self-examination (MSE)	Scott et al 2010 (UK)<br>Elango et al. 2011 (India)<br>Jornet et al. 2015 (Spain)<br>Viswanath et al. 2013 (US)<br>Chaudhari et al. 2013 (India)<br>Ghani et al. 2019 (Malaysia)

Supplemental Table 3. Cost-effectiveness of oral cancer screening - Studies based on visual oral screening

Author/Year (Country)	Economic evaluation	Comparison	Measures	Results	Conclusion
**Community Screening studies- real world data**					
Subramanian et al. 2009 (India)	Cost-effectiveness	Screening trial (intervention) vs No screening (control)	Cost per life-year saved by performing intervention	US$ 835 for all individuals<br>US$ 156 for high-risk individuals.	Screening the high-risk population is cost-effective.
Huang et al. 2019 (Taiwan)	Cost-effectiveness	Data from national screening program (intervention) vs Data from cancer registry (control)	Cost per life-year saved by performing intervention	US$ 28,516 for all individuals<br>US$ 5579 for individuals detected up to stage 1.	Screening is cost effective if oral cancer detected up to stage 1, and cost saving if patient detected as an OPMD.
**Modelling studies of health economic evaluation**					
Speight et al. 2006 (UK)	Cost-utility analysis	7 screening models vs No screening	Cost per life-year saved by performing intervention	£ 18,919 for opportunistic high-risk screening of individuals by a GDP or FP	High-risk opportunistic screening by a GDP or FP may be cost-effective.
Dedhia et al. 2011 (USA)	Markov model	Annual community screening vs No screening	QALYs	Screen cohort gains an average of 0.0151 QALYs	Community-based screening program targeting high-risk males is likely to be cost-effective.
Vokó et al. 2016 (Hungary)	Markov model Cost-utility analysis	2 screening models Vs No screening	QALYs and ICER		High-risk opportunistic screening is more cost-effective than organized screening and no screening.
van der Meij et al. 2002 (Netherlands)	Cost-utility analysis	Screening for oral cancer in OLP patients vs No screening	Cost per life-year saved by performing intervention	US$ 53,400 for all individuals with OLP	Screening for oral cancer in OLP patients seems attractive, if malignant transformation rate is <0.4%/year
Kumdee et al. 2018 (Thailand)	Markov model Cost-utility analysis	Four screening models vs No screening	Cost per life-year saved by performing intervention<br><br>QALYs	THB 1362 for all screened individuals<br><br>Screen cohort gains 0.0044 QALYs	Screening in patients >40 was not cost-effective.

Quality-adjusted Life years (QALY); incremental cost-effectiveness ratios (ICER); Equivalent life saved (ELS); General dental practitioner / Family practitioner (GDP / FP). Thai bhat (THB)

Supplemental Table References:
Axéll T (1976). A prevalence study of oral mucosal lesions in an adult Swedish population. Odontol Revy Suppl 36:1-103.
Chang IH, Jiang RS, Wong YK, Wu SH, Chen FJ, Liu SA (2011). Visual screening of oral cavity cancer in a male population: Experience from a medical center. Journal of the Chinese Medical Association: JCMA 74(12):561-566.
Chaudhari A, Hegde-Shetiya S, Shirahatti R, Agrawal D (2013). Comparison of different screening methods in estimating the prevalence of precancer and cancer amongst male inmates of a jail in Maharashtra, India. Asian Pac J Cancer Prev 14(2):859-864.
Chuang SL, Su WW, Chen SL, Yen AM, Wang CP, Fann JC et al. (2017). Population-based screening program for reducing oral cancer mortality in 2,334,299 Taiwanese cigarette smokers and/or betel quid chewers. Cancer 123(9):1597-1609.
Dedhia RC, Smith KJ, Johnson JT, Roberts M. The cost-effectiveness of community-based screening for oral cancer in high-risk males in the United States: a Markov decision analysis approach. Laryngoscope 2011;121:952-60.
Dombi C, Czeglédy A, Gyurkovics C, Freisleben A, Sári K (1994). [stomatologic mass screening in the 3rd district of Budapest]. Fogorv Sz 87(2):45-48.
Elango KJ, Anandkrishnan N, Suresh A, Iyer SK, Ramaiyer SK, Kuriakose MA (2011). Mouth self-examination to improve oral cancer awareness and early detection in a high-risk population. Oral Oncol 47(7):620-624.
Feaver GP (1997). Screening for oral cancer and precancer. - the industrial experience. Oral Dis 3 (Sup 2):535
Fernandez Garrote L, Sankaranarayanan R, Lence Anta JJ, Rodriguez Salva A, Maxwell Parkin D (1995). An evaluation of the oral cancer control program in Cuba. Epidemiology (Cambridge, Mass) 6(4):428-431.
Ghani WMN, Razak IA, Doss JG, Ramanathan A, Tahir Z, Ridzuan NA, Edgar S, Zain RB (2019). Mouth self-examination as a screening tool for oral potentially malignant disorders among a high-risk indigenous population. J Public Health Dent 79(3):222-230
Harris CK, Warnakulasuriya KA, Cooper DJ, Peters TJ, Gelbier S (2004). Prevalence of oral mucosal lesions in alcohol misusers in south london. J Oral Pathol Med 33(5):253-259.
Huang CC, Lin CN, Chung CH, Hwang JS, Tsai ST, Wang JD. Cost-effectiveness analysis of the oral cancer screening program in Taiwan. Oral Oncol 2019;89:59-65.
Jornet PL, Garcia FJ, Berdugo ML, Perez FP, Lopez AP (2015). Mouth self-examination in a population at risk of oral cancer. Aust Dent J 60(1):59-64.
Kumdee C, Kulpeng W, Teerawattananon Y. Cost-utility analysis of the screening program for early oral cancer detection in Thailand. PLoS One 2018;13:e0207442.
Lim K, Moles DR, Downer MC, Speight PM (2003). Opportunistic screening for oral cancer and precancer in general dental practice: Results of a demonstration study. Br Dent J 94(9):497-502; discussion 493
Mehta FS, Gupta PC, Bhonsle RB, Murti PR, Daftary DK, Pindborg JJ (1986). Detection of oral cancer using basic health workers in an area of high oral cancer incidence in india. Cancer Detection and Prevention 9(3-4):219-225.
Jullien JA, Downer MC, Zakrzewska JM, Speight PM (1995). Evaluation of a screening test for the early detection of oral cancer and precancer. Community dental health 12(1):3-7.
Mathew B, Sankaranarayanan R, Sunilkumar KB, Kuruvila B, Pisani P, Nair MK (1997). Reproducibility and validity of oral visual inspection by trained health workers in the detection of oral precancer and cancer. Br J Cancer 76(3):390-394.
Monteiro LS, Salazar F, Pacheco JJ, Martins M, Warnakulasuriya S (2015). Outcomes of invitational and opportunistic oral cancer screening initiatives in Oporto, Portugal. J Oral Pathol Med 44(2):145-152
Nagao T, Ikeda N, Fukano H, Miyazaki H, Yano M, Warnakulasuriya S. 2000. Outcome following a population screening programme for oral cancer and precancer in Japan. Oral Oncol 36(4):340-346.
Nagao T, Warnakulasuriya S, Gelbier S, Yuasa H, Tsuboi S, Nakagaki H (2003). Oral pre-cancer and the associated risk factors among industrial workers in Japan's overseas enterprises in the UK. J Oral Pathol Med 32(5):257-264.
Psoter WJ, Morse DE, Kerr AR, Tomar SL, Aguilar ML, Harris DR et al. (2019). Oral cancer examinations and lesion discovery as reported by U.S. general dentists: Findings from the national dental practice-based research network. Prev Med 124:117-123.
Sankaranarayanan R, Mathew B, Jacob BJ, Thomas G, Somanathan T, Pisani P et al. (2000). Early findings from a community-based, cluster-randomized, controlled oral cancer screening trial in Kerala, India. The Trivandrum oral cancer screening study group. Cancer 88(3):664-673.

Scott SE, Rizvi K, Grunfeld EA, McGurk M (2010). Pilot study to estimate the accuracy of mouth self-examination in an at-risk group. Head Neck 32(10):1393-1401.
Speight PM, Palmer S, Moles DR, Downer MC, Smith DH, Henriksson M, Augustovski F. The cost-effectiveness of screening for oral cancer in primary care. Health Technol Assess 2006;10:1-144, iii-iv.
Subramanian S, Sankaranarayanan R, Bapat B, Somanathan T, Thomas G, Mathew B, Vinoda J, Ramadas K. Cost-effectiveness of oral cancer screening: results from a cluster randomized controlled trial in India. Bull World Health Organ 2009;87:200-6.
Talamini R, Barzan L, Franceschi S, Caruso G, Gasparin A, Comoretto R (1994). Determinants of compliance with an early detection programme for cancer of the head and neck in north-eastern Italy. Eur J Cancer B Oral Oncol 30b(6):415-418.
van der Meij EH, Bezemer PD, van der Waal I. Cost-effectiveness of screening for the possible development of cancer in patients with oral lichen planus. Community Dent Oral Epidemiol 2002;30:342-51.
Viswanath A, Kerns TJ, Sorkin JD, Dwyer DM, Groves C, Steinberger EK (2013). Self-reported oral cancer screening by smoking status in Maryland: Trends over time. J Public Health Dent 73(4):261-270.
Vokó Z, Túri G, Zsólyom A. A szájüregi szűrés költséghatékonysága Magyarországon [Cost-effectiveness of oral cancer screening in Hungary]. Orv Hetil. 2016;157:1161-70. (Hungarian).
Warnakulasuriya KA, Ekanayake AN, Sivayoham S, Stjernsward J, Pindborg JJ, Sobin LH, Perera KS. (1984). Utilization of primary health care workers for early detection of oral cancer and precancer cases in Sri Lanka. Bull World Health Org 62(2):243-250.
Warnakulasuriya KA, Nanayakkara BG (1991). Reproducibility of an oral cancer and precancer detection program using a primary health care model in Sri Lanka. Cancer Ddetection and Prevention 15(5):331-334
Warnakulasuriya S, Kashyap R, Dasanayake AP (2010). Is workplace screening for potentially malignant oral disorders feasible in india? J Oral Pathol Med 39(9):672-676.
